# P-1634. Prescribing Patterns of Antibiotics in an Academic Medical Center Internal Medicine Clinic

**DOI:** 10.1093/ofid/ofae631.1800

**Published:** 2025-01-29

**Authors:** Michael Dickens, Brady Woods, Sara Al Azmeh, Erin Weslander

**Affiliations:** Northwestern Medicine, Chicago, Illinois; Northwestern Memorial Hospital, Bolivar, Ohio; Northwestern Memorial Hospital, Chicago, Illinois; Northwestern Memorial Hospital, Bolivar, Ohio

## Abstract

**Background:**

Antimicrobial stewardship is critical in the prevention of antibiotic resistance. The CDC estimates nearly 90% of antibiotic use occurs in outpatient settings, with up to half of the prescriptions not carrying appropriate indications. Public health efforts like the National Action Plan for Combating Antibiotic Resistant Bacteria aim to reduce/improve outpatient antibiotic use following the Department of Health and Human Services guidance.

Inclusion/Exclusion

**Figure:**
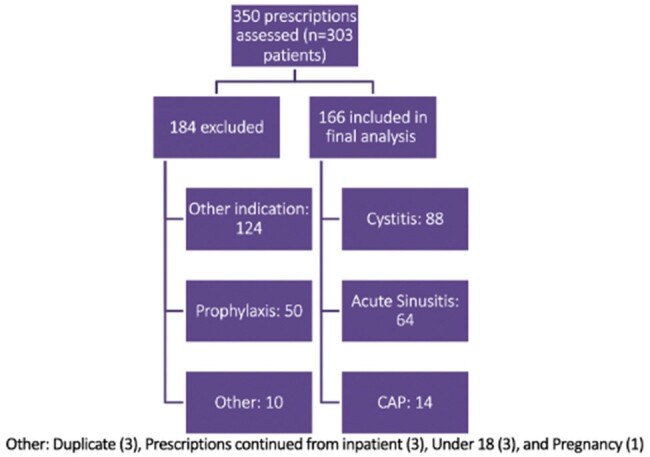

350 prescriptions were assessed for inclusion within the study. Prescriptions were excluded if antibiotics were prescribed for prophylaxis, indications outside of preferred list (notably: COPD Exacerbations, prostatitis, pyelonephritis, clearly documented “asymptomatic bacteriuria”), prescriptions continued from inpatient admission, pregnancy, under the age of 18, and infectious disease consults

**Methods:**

A retrospective descriptive assessment of antimicrobial prescribing practice within one internal medicine clinic was conducted through chart review. Outpatient antibiotic prescriptions from a prespecified list of agents written between March to June of 2023 for cystitis, community-acquired pneumonia, and acute sinusitis were compared to current Infectious Disease Society of America recommendations for treatment. The primary outcome was appropriateness based on indication, drug choice, dose, and duration of therapy. A key secondary outcome assessed was ED or clinic return visits for the same primary problem within 30 days.

Prescription Appropriateness

**Figure:**
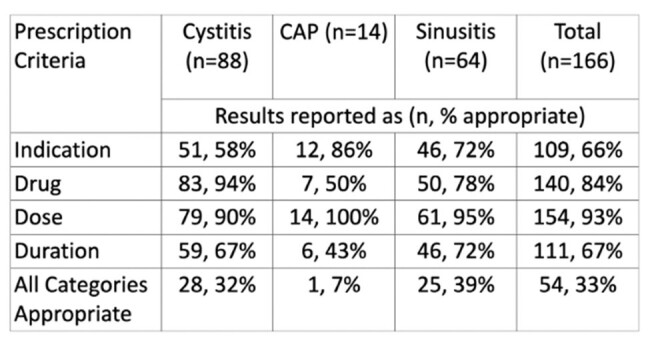

Each prescription was assessed for correct indication, drug, dose, and duration based on current IDSA guidelines.

**Results:**

This study included 166 antibiotic prescriptions written for 147 patients. Of these prescriptions, 67% were inappropriately prescribed. Duration and indication were inappropriate most frequently on prescriptions while drug and dose were appropriately selected by providers. Of the patients with prescription errors, 23.1% (25/108) led to a return visit versus 17.2% (10/58) for patients with antibiotics prescribed correctly (p =0.429).

Prescription Inappropriateness by Type of Error

**Figure:**
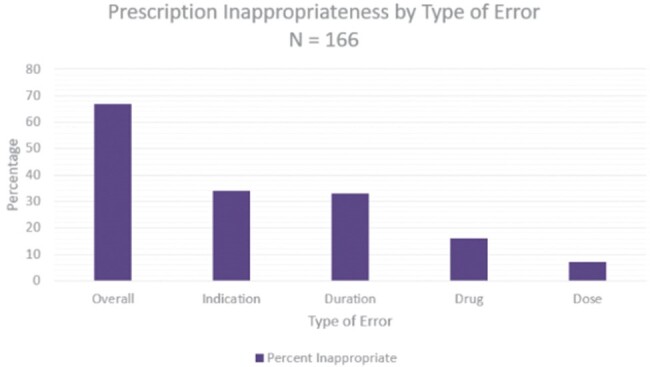

This study included 166 antibiotic prescriptions written for 147 patients. Of these prescriptions, 67% were inappropriately prescribed. Duration (33%) and indication (34%) were inappropriate most frequently on prescriptions while drug (16%) and dose (7%) were appropriately selected by providers.

**Conclusion:**

In conclusion, antibiotics are inappropriately prescribed in the outpatient setting which necessitates the need for increased stewardship efforts. A trend towards increased return visits was observed for patients who received an inappropriate antibiotic prescription. As a secondary outcome, this study was unable to meet statistical significance, but further evaluation should be done to explore this trend. Implementation of institutional prescribing guidelines for common infections such as acute sinusitis or cystitis paired with targeted education for outpatient clinics could help improve antimicrobial prescribing habits.

Antibiotic Prescription by Appropriateness

**Figure:**
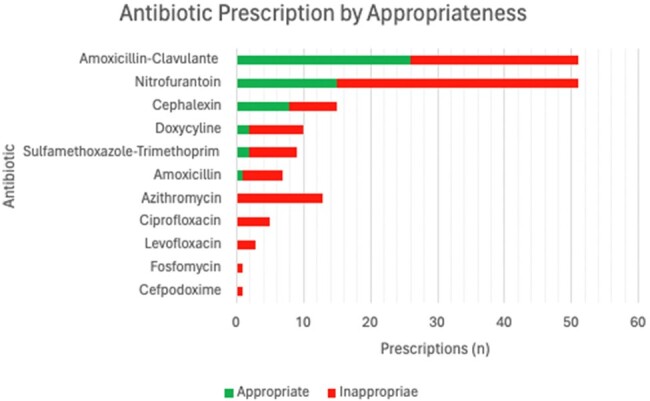

The most common antibiotics prescribed included amoxicillin-clavulanate, cephalexin, and nitrofurantoin accounting for 70% of the prescriptions reviewed. Fluroquinolones when prescribed were inappropriate in this study based on indication, dose, duration, or drug. Amoxicillin-clavulanate when prescribed was correct in all four categories for only 50% of prescriptions while nitrofurantoin was appropriate only 29% of the time.

**Disclosures:**

**All Authors**: No reported disclosures

